# Comparative analysis of the intestinal microbiota of black−necked cranes (*Grus nigricollis*) in different wintering areas

**DOI:** 10.3389/fcimb.2023.1302785

**Published:** 2024-01-22

**Authors:** Zhongbin Wang, Erhao Zhang, Ying Tang, Jiujiu Wu, Suliman Muhammad, Peng Shang, Cheng Zong, Ke Rong, Jianzhang Ma

**Affiliations:** ^1^ College of Wildlife and Protected Area, Northeast Forestry University, Harbin, China; ^2^ Department of Resources and Environment, Tibet Agricultural and Animal Husbandry College, Linzhi, Tibet, China

**Keywords:** fecal microbiota, Grus nigricollis, Tibet, bacteria, fungi

## Abstract

Fecal microbiota is essential for host health because it increases digestive effectiveness. The crane species *Grus nigricollis* (*G. nigricollis*) is considered to be near threatened. The fecal microbial composition of crane is less understood, particularly in the Tibet, China. This study was performed to investigate the differences in fecal microbial composition and diversity of crane in different wintering areas using third-generation single-molecule real-time sequencing technology in the Tibet, China. According to the findings, 20 samples were used to generate 936 bacterial amplicon sequence variants (ASVs) and 1,800 fungal ASVs, only 4 bacterial ASVs and 20 fungal ASVs were shared in four distinct locations. Firmicutes were the dominant bacterial phylum in all samples, and Ascomycota and Basidiomycota were the dominant fungal phylum. At the genus level, *Lactobacillus* was the dominant genus in Linzhi City (LZ), Shannan City (SN), and Lasa City (LS), whereas Megamonas was the dominant genus in Rikaze City (RKZ). *Naganishia* and *Mycosphaerella* were the dominant fungal genera in SN and RKZ. *Mycosphaerella* and *Tausonia* were the dominant fungal genera in LZ. *Naganishia* and *Fusarium* were the dominant fungal genera in LS. And the fecal microbial composition varied between the four groups, as shown by the underweighted pair-group method with arithmetic means and principal coordinates analysis. This study offers a theoretical basis for understanding the fecal microbial composition of crane.

## Introduction

The black-necked crane (*Grus nigricollis* [*G. nigricollis*]) is a unique, rare crane species and a Class I protected animal in China. There are 15 species of crane in the world, but only the black-necked crane is a plateau crane. The northern and western parts of the Qinghai–Tibet Plateau are its breeding areas. The main wintering areas are the Yarlung Zangbo River valley, the southern Himalayan slope, and the Yunnan Guizhou Plateau. Black-necked cranes can only reproduce and spend the winter in Tibet. Therefore, protecting black-necked cranes in Tibet is essential to their continued existence.

Animal gut microbiota are critical in individual nutrient absorption, metabolic regulation, and immune function. They also play a crucial role in maintaining the organism’s health and adaptive evolution (Waite and Taylor, 2015; Gao et al., 2016; Li et al., 2018). Birds serve as a critical environmental indicator organism with their diverse genetic makeup and species diversity. The study of the composition and function of the intestinal microbiota of birds has increased exponentially in recent years due to the development of molecular biology techniques and growing interest in the bird population (Dong et al., 2021; Wang et al., 2021). However, due to the limitations of sampling challenges and low DNA extraction amounts in field conditions, the research was primarily focused on poultry, and there were relatively few studies on the intestinal microbiota of wild birds (Gao et al., 2016). The physiological activities of birds are subject to more substantial selective pressure due to their complex life history characteristics, diverse feeding habits, mating system, physiological characteristics, flying life, long-distance migration, etc., which complicate changes in the intestinal microbiota. The species, environment, life-cycle stage, digestive tract region, and other factors impact the composition and diversity of the intestinal microbiota of birds (Sun et al., 2022). The formation of the intestinal microbiota in birds is mainly influenced by food composition (Youngblut et al., 2018). The composition and essential functions of enterobacteria have been reported to play an important role in maintaining host homeostasis. However, the intestinal microbiota of the black-necked crane in the Tibet Autonomous Region is unknown. Here, we used a third-generation single-molecule real-time sequencing technology to characterize the fecal microbial community and compare the differences in fecal microbial composition and diversity of cranes living in four different geographical regions in Tibet, China.

## Materials and methods

### Sample collection

Fecal samples were collected from cranes living in four different regions: Linzhi City (LZ), Lasa City (LS), Shannan City (SN), and Rikaze City (RKZ), Tibet, China. The geographical sources and sample codes are shown in **Table 1**. At each sample location, 20 fresh fecal samples were collected in the morning and thoroughly mixed to form a single composite sample to ensure the experiment was representative. Using sterile disposable forceps to remove the surface, only the middle portions of the fecal samples were collected to avoid contamination from the ground. The samples were then stored in 15 mL sterile centrifuge tubes. The samples were immediately placed in a −20°C portable freezer and stored at −80°C for long-term preservation.

**Table 1:** Geographical sources and sample codes.

Sampling Site	Linzhi City	Lasa City	Shannan City	Rikaze City
Habitat type	Nunja	Nunja	Nunja	Nunja
Latitude	29°21′42.7′′N	29°51′31.4′′N	29°17′22.5′′N	29°19′40.3′′N
Longitude	94°25′28.5′′E	91°21′36.5′′E	91°7′4.1′′E	88°49′23.1′′E
Altitude (m)	2 917	3 705	3 560	3 813
Annual average temperature (°C)	9.30	2.90	8.60	6.30
Annual average precipitation (mm)	652.60	491.00	356.60	400.00
Main food	Grain residues	Grain residues	Grain residues and insects	Plant roots, stems, leaves, and insects

### Fecal-sample DNA extraction, amplification, and MiSeq sequencing

According to the manufacturer’s instructions, the total DNA was extracted using an OMEGA-soil kit (Omega Bio-Tek, United States, Cat. # D5625-01). The purity and concentration of DNA were assessed using a NanoDrop ND-2000 ultraviolet spectrophotometer (Thermo Scientific, Wilmington, United States), and the DNA quality was determined using 0.8% agarose gel electrophoresis. The bacterial 16S rRNA gene sequence was amplified using the primers 27F (5′-AGAGTTTGATCMTGGCTCAG-3′) and 1492R (5′-ACCTTGTTACGACTT-3′) (Yang et al., 2018) and the fungal internal transcribed spacer (ITS) sequence was amplified using the primers ITS1F (5′-CTTGGTCATTTAGAGGAAGTAA-3′) and ITS4R (5′-TCCTCCGCTTATTGATATGC-3′) (Shi et al., 2021). A total volume of 25 μL was used for the polymerase chain reaction (PCR), which contained 12.5 μL of 2X Taq PCR MasterMix, 1 μL of forward and reverse primers (10 mM), 1 μL of DNA template, and 9.5 μL of distilled deionized water. The following conditions were used for the PCR amplification: initial denaturation at 95°C for 5 min, followed by 35 cycles of denaturation at 95°C for 30 s, annealing at 58°C for 30 s, and extension at 72°C for 90 s; 72°C for 10 min. The electrophoresis of 1% agarose gel was used to monitor the PCR product, which was purified using the MinElute PCR purification kit (Qiagen) and quantified using the Qubit fluorometer (Invitrogen, Waltham, MA, USA). The purified amplicons were sequenced using a PacBio-based single-molecule real-time (SMRT) sequencing platform (Pacific Biosciences, Menlo Park, CA, USA) from Personal Biotechnology Co., Ltd. (Shanghai, China).

### Data processing and analysis

The PacBio Sequel II platform generated the raw sequencing reads. The high-quality sequences were obtained through denoising and filtering using the DADA2 package (version 1.16) in R (version 4.0.3) and Vsearch software (version 2.13.4) (Callahan et al., 2016; Rognes et al., 2016). After quality was controlled, the optimized sequences were transferred into QIIME2 software (version 2019.10) for additional downstream analysis (Bolyen et al., 2019). Amplicon sequence variants (ASVs) were clustered using Uparse software (version 7.1) and a cutoff of >97% (Edgar, 2013). Then, using the Greengenes database (Release 13.8, http://greengenes.secondgenome.com/) for the bacterial community (Quast et al., 2013) and the UNITE database (Release 8.0, https://unite.ut.ee/) for the fungal community (Koljalg et al., 2013), the ASVs were annotated and assigned taxonomies by QIIME2 software (version 2019.10).

### Statistical analysis

The Chao1 index, Shannon index, and Simpson index were among the alpha diversity indices of microbiota calculated by QIIME2 and the ggplot2 package of the R project. Alpha diversity indices between certain groups were evaluated using the Kruskal–Wallis test with multiple testing corrections. Venn diagram of software R drew Venn diagrams based on the ASVs sequence to display the number of standard and unique ASVs. A principal component analysis based on Bray–Curtis distance was generated using QIIME2 and the R project ape package to analyze community dissimilarity. Heatmap and hierarchical clustering were sketched using R tools, and the linear discriminant analysis (LDA) effect size was used to assess the specific species differences (Segata et al., 2011).

## Results

### Sequencing-data analysis

**Figure 1:**
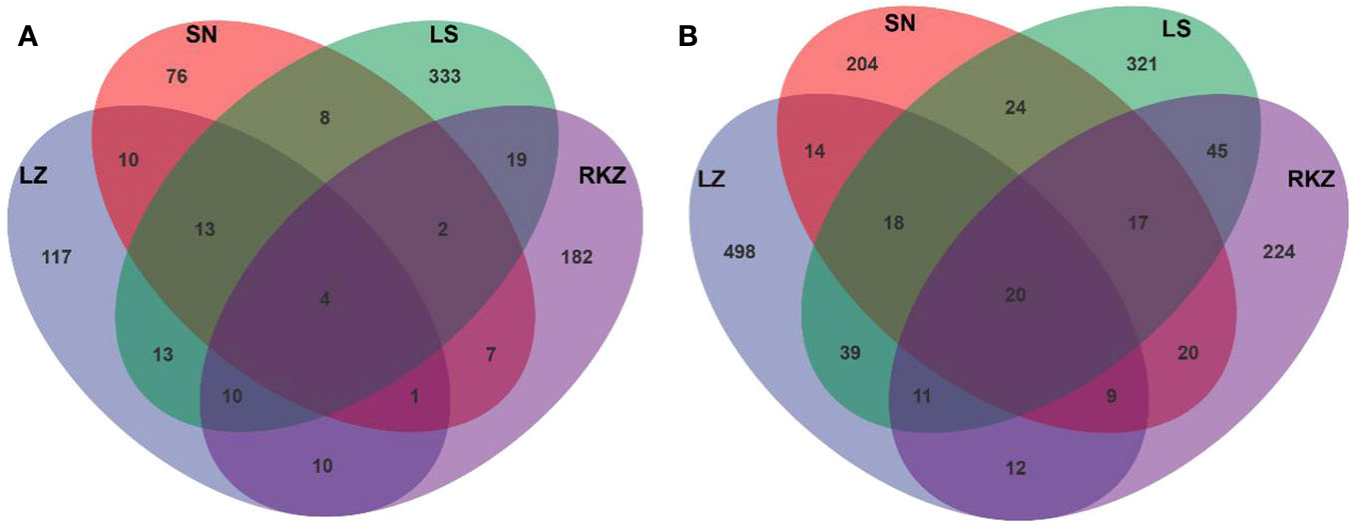
Venn diagrams of bacterial **(A)** and fungal **(B)** amplicon sequence variants detected in the fecal samples of crane.

Crane fecal samples yielded 46,460 effective bacterial sequences after quality control, ranging from 9,925 to 13,778 per sample. The length of each effective sequence ranged from 1,385 bp to 1,819 bp, with an average length of 1,476 bp (**Table 2**). All samples found 60,979 effective fungal sequences, ranging from 12,089 bp to 22,959 bp per sample. The length of each effective sequence ranged from 654 bp to 2,800 bp long, with an average length of 1,209 bp (**Table 2**). Sequencing coverage and rarefaction curves were used to assess the sequencing depth. The rarefaction curves analysis showed that the sob index reached saturation with increasing sequencing depth, reflecting the actual number of observed species in the samples (**Figure S1**), indicating that the sequencing data was sufficient and could reflect the existing microbial community in the samples. The sequencing coverage was >99% per sample. After classification matching, 936 bacterial ASVs and 1,800 fungal ASVs were obtained from all samples, with the LZ, SN, LS, and RKZ groups generating 178, 121, 402, and 235 bacterial ASVs, respectively, and 621, 326, 495, and 358 fungal ASVs, respectively. Only 20 fungal ASVs were shared, according to 1.11% of the total fungal ASVs, and only four bacterial ASVs were shared in four distinct locations, according to 0.43% of the total bacterial ASVs, based on the Venn diagram at the ASV level (**Figures 1A, B**). These findings showed a significant difference in the microbial community composition in the feces of cranes from four different regions.

**Table 2:** Amplicon sequence variants and related sequence indexes in the fecal samples of crane.

Samples	Effective sequence number		Average length/bp		Sequencing coverage/%		Number of ASVs	
	Bacteria	Fungi	Bacteria	Fungi	Bacteria	Fungi	Bacteria	Fungi
LZ	9 925	12 089	1 476	1 209	99.98	99.92	178	621
SN	12 433	13 707	1 476	1 209	99.99	99.93	121	326
LS	10 324	12 224	1 476	1 209	99.91	99.77	402	495
RKZ	13 778	22 959	1 476	1 209	99.98	99.82	235	358

### Alpha diversity analysis

**Figure 2:**
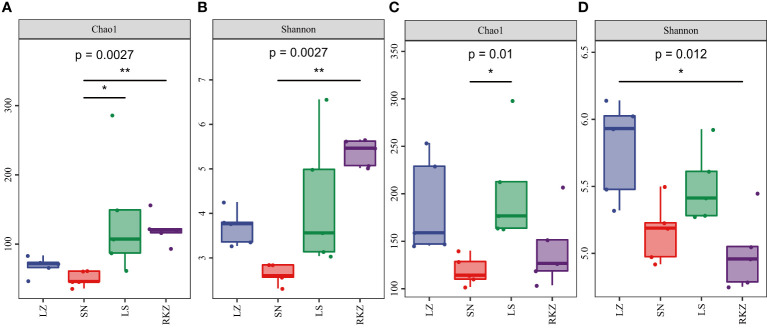
Alpha diversity indexes of the bacterial **(A, B)** and the fungal **(C, D)** communities in the fecal samples of crane. * represents P < 0.05 and ** represents P < 0.01.

The alpha diversity was calculated by QIIME2 and R project ggplot2 package, including Chao1 and Shannon index, to analyze further the changes in the fecal microbial community of cranes living in different habitats. The results revealed a significant difference between the groups, with LS and RKZ having higher community richness (Chao1 index) of bacterial species and SN having the lowest (**Figure 2A**). When comparing the bacterial community diversity of the three groups, RKZ exhibited the highest Shannon index. (**Figure 2B**). LS had higher community richness (Chao1 index) of fungal species than SN, which was significantly different (*p* = 0.017). However, no significant differences existed among the other habitats (**Figure 2C**). The Shannon index for the fungal community diversity in LZ was highest, and that in RKZ was lowest, significantly different between LZ and RKZ (*p* = 0.017). Nevertheless, other habitats had no significant difference (**Figure 2D**). In conclusion, the alpha diversity in the fecal microbial community of cranes inhabiting four geographical regions differed.

### The fecal microbial community composition

**Figure 3:**
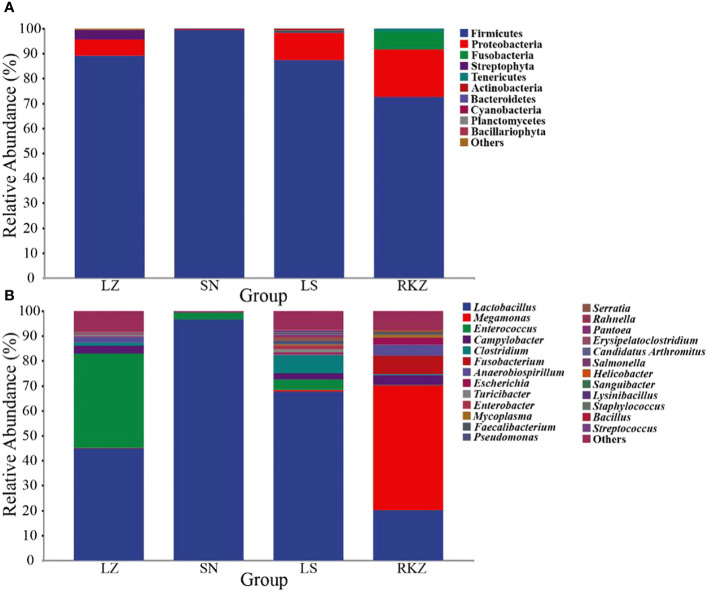
Composition of the bacterial community in the fecal samples of the crane at the phylum **(A)** and the genus **(B)** levels.

**Figure 4:**
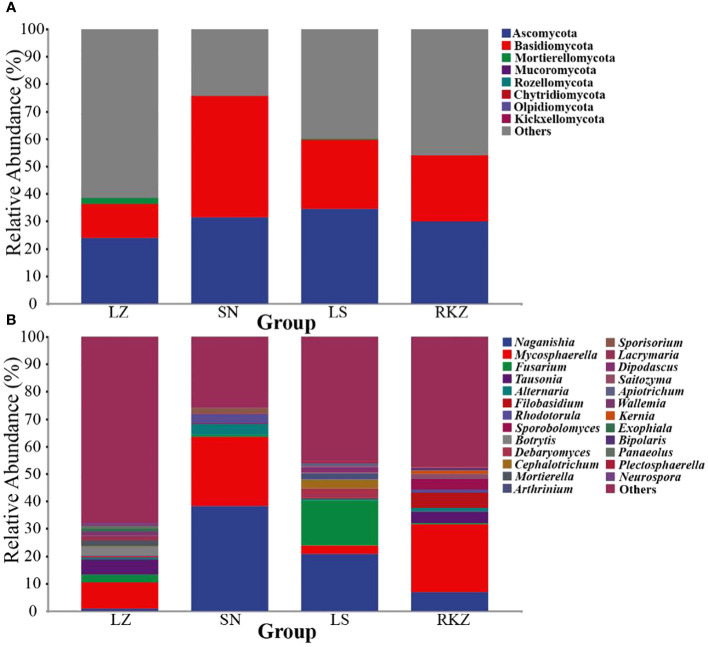
Composition of the fungal community in the fecal samples of the crane at the phylum **(A)** and the genus **(B)** levels.

**Figure 5:**
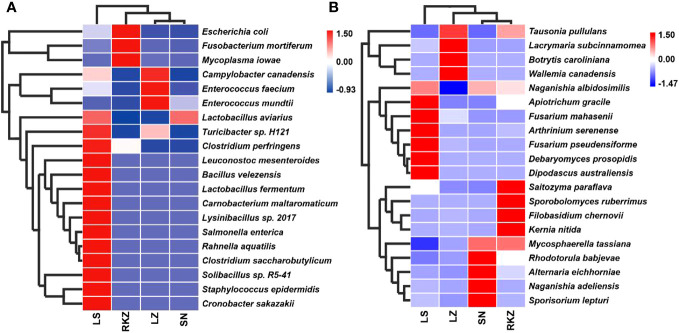
Composition of the bacterial **(A)** and the fungal **(B)** communities in the fecal samples of crane at the species levels.

The bacterial ASVs were classified into 10 phyla, 22 classes, 39 orders, 109 families, 109 genera, and 129 species across all fecal samples. The fungal ASVs were classified into 9 phyla, 22 classes, 43 orders, 82 families, 125 genera, and 183 species. The fecal microbial composition was analyzed at the phylum and the genus levels. The dominant bacterial phyla in the four groups of the crane were Firmicutes (89.11% in LZ, 99.47% in SN, 87.35% in LS, and 72.56% in RKZ), Proteobacteria (6.37%), and Streptophyta (3.83%) were the second dominant phyla in LZ; Proteobacteria (10.79%) was the second dominant phyla in LS; Proteobacteria (19.04%), Fusobacteria (7.33%), and Tenericutes (1.04%) were the second dominant phyla in RKZ. Except for Firmicutes, no phyla demonstrated an average relative abundance >1% in SN. The findings showed that the bacterial communities in feces from different geographical regions differed (**Figure 3A**). At the genus level, 15 bacterial genera showed an average relative abundance >1% in all fecal samples, namely *Enterococcus*, *Lactobacillus*, *Clostridium*, unclassified_f_Lachnospiraceae, unclassified_f_Peptostreptococcaceae, *Faecalibacterium*, *Megamonas*, *Fusobacterium*, unclassified_o_Burkholderiales, *Campylobacter*, *Anaerobiospirillum*, *Escherichia*, *Rahnella*, *Serratia*, and *Pseudomonas*. *Lactobacillus* was the dominant genus in LZ (44.90%), SN (96.72%), and LS (67.69%), whereas *Megamonas* was the dominant genus in RKZ (49.84%). Meanwhile, the composition and relative abundance of the bacterial communities differed between groups (**Figure 3B**). The dominant fungal phyla in the four groups (LZ, SN, LS, and RKZ) of the crane were Ascomycota (23.85%, 31.29%, 34.53%, and 29.83%, respectively) and Basidiomycota (12.35%, 44.29%, 25.29%, and 24.23%, respectively) (**Figure 4A**). As shown in **Figure 4B**, at the genus level, 18 fungal genera showed an average relative abundance >1% in the fecal sample, namely *Mycosphaerella*, unclassified_Didymellaceae, *Alternaria*, *Exophiala*, *Botrytis*, *Debaryomyces*, *Dipodascus*, *Fusarium*, *Cephalotrichum*, *Lacrymaria*, *Rhodotorula*, *Sporobolomyces*, *Tausonia*, *Filobasidium*, *Naganishia*, *Saitozyma*, *Sporisorium*, and *Mortierella*. *Naganishia* (38.22% and 6.84%) and *Mycosphaerella* (25.16% and 24.78%) were the dominant fungal genera in SN and RKZ. *Mycosphaerella* (9.54%) and *Tausonia* (5.63%) were the dominant fungal genera in LZ. *Naganishia* (20.80%) and *Fusarium* (16.19%) were the dominant fungal genera in LS. The findings showed differences in the group composition and relative abundance of the dominating fungal genera. Further analysis of the heatmap of microbial community compositions at the species level (**Figure 5**) indicated that *Campylobacter canadensis*, *Enterococcus faecium*, *Lactobacillus aviaries*, *Fusobacterium mortiferum*, *Escherichia coli*, *Turicibacter* sp. H121, and *Rahnella aquatilis* were the dominant bacterial species, with 20 fungal species showing an average relative abundance >1% in all samples, for example, *Mycosphaerella tassiana*, *Naganishia albidosimilis*, and *Naganishia adeliensis*. However, the dominant microbial species per sample was different.

### Beta diversity analysis

**Figure 6:**
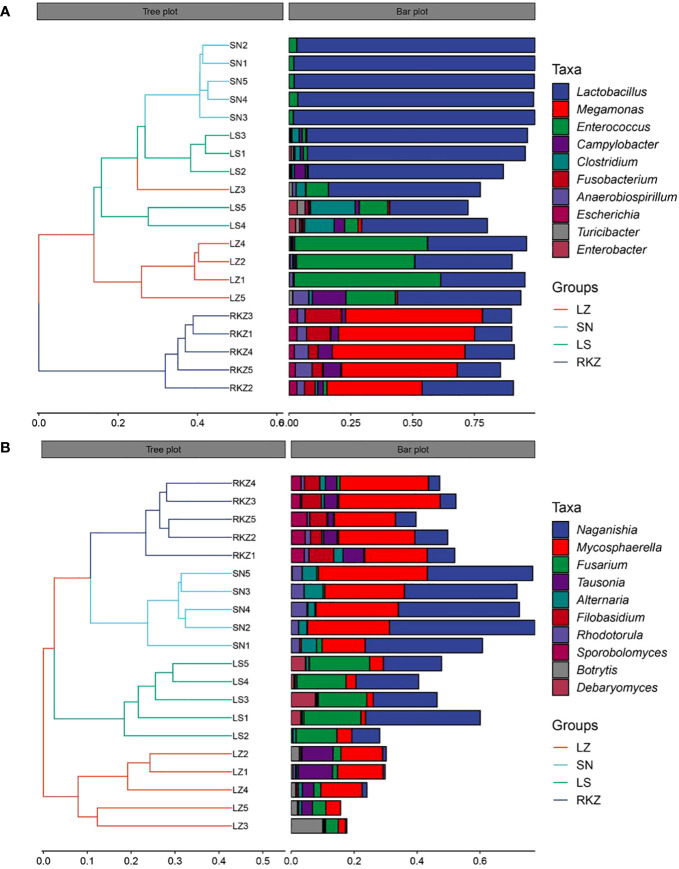
Hierarchical clustering of the fecal bacterial **(A)** and fungal **(B)** based on the unweighted pair-group method with arithmetic means at the genus level.

**Figure 7:**
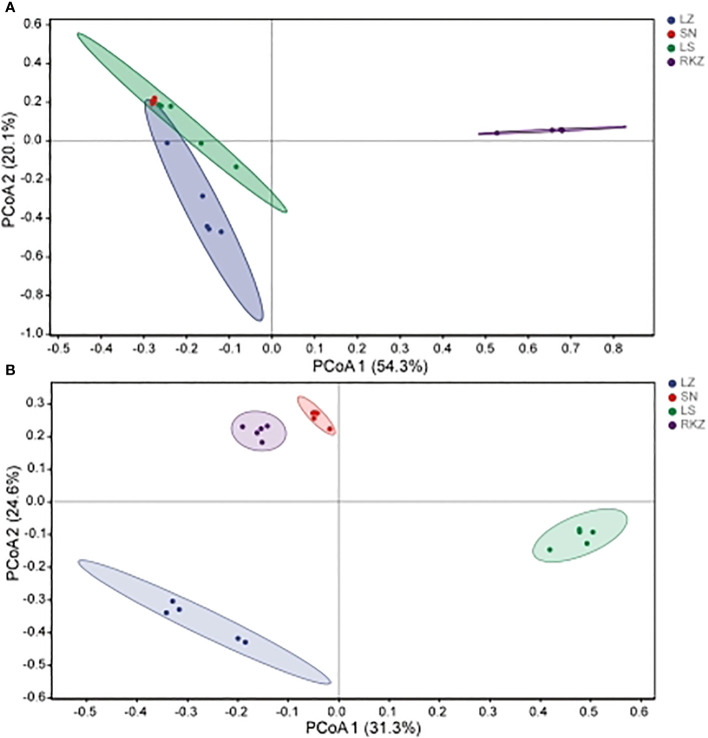
Principal component analysis of the bacterial **(A)** and the fungal **(B)** communities in the fecal samples of crane.

The beta diversity analysis, which included hierarchical clustering based on an unweighted pair-group method with arithmetic means at the genus level and principal coordinates analysis (PCoA) based on Bray-Curtis distances, was conducted to explore further the dissimilarity in fecal microbial community composition among various groups. The feces from the same group were significantly clustered, according to the results of the hierarchical clustering analysis, suggesting that the composition of the fecal microbial community was very similar. Comparatively, the bacterial community of all samples was divided into three groups, with the SN and LS constituting the first cluster, LZ the second cluster, and RKZ the third cluster (**Figure 6A**). This was in contrast to the fungal community, where the RKZ and SN constituted the first cluster, LS the second cluster, and LZ the third cluster (**Figure 6B**). The PCoA was used to analyze and further display the statistical difference. As shown in **Figure 7A**, at the genus level, the different explaining rate of PCoA1 and PCoA2 was 54.3% and 20.1%, respectively, with a sum of 74.4% (**Figure 7A**), which separated the bacterial samples into three groups that matched the results of hierarchical clustering analysis. The different explaining rate of PCoA1 and PCoA2 in the fungal samples was 31.3% and 24.6%, respectively, with a sum of 55.9% (**Figure 7B**) that separated the fungal samples into four groups. The results of PCoA were similar to hierarchical clustering analysis. These results suggested that various groups had diverse fecal microbial compositions.

### Differences in fecal microbial communities between different groups

**Figure 8:**
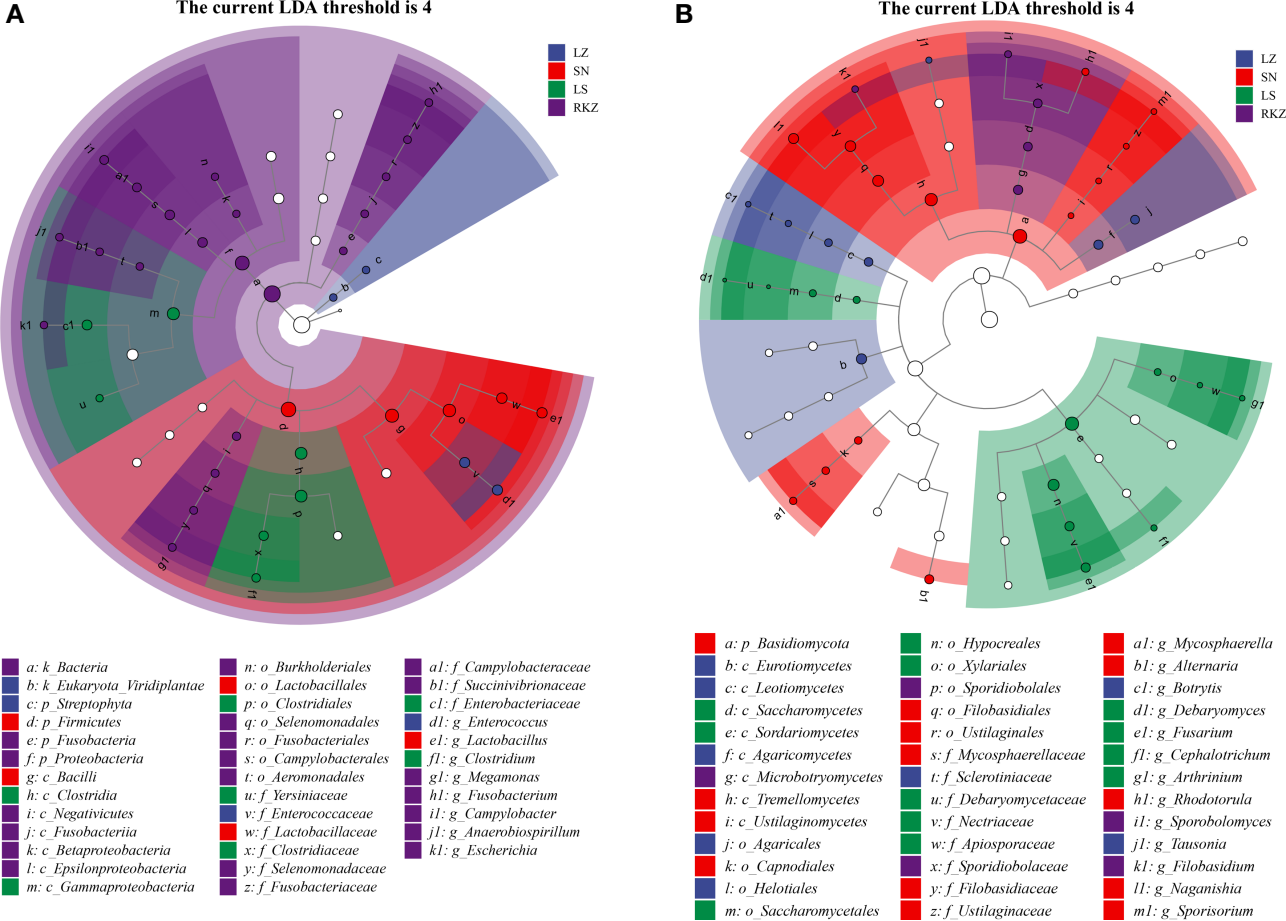
Linear discriminant analysis effect size analysis showing the fecal bacterial **(A)** and fungal **(B)** biomarkers with significant differences in the fecal samples of crane (linear discriminant analysis score >4.0).

**Figure 9:**
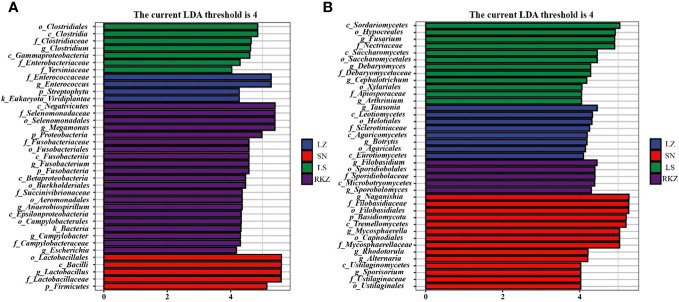
The plot from linear discriminant analysis effect size analysis on the fecal bacterial **(A)** and fungal **(B)** biomarkers of crane living in different geographical regions (linear discriminant analysis score >4.0).

Linear discriminant analysis effect size (LEfSe), based on an LDA score >4.0, was performed to visualize the significant difference in fecal microbes of cranes between four geographical regions (**Figure 8**). We screened the biomarker taxa among different groups (**Figure 9**). According to the LEfSe analysis plot, 37 bacterial characteristics were significantly other among the LZ, SN, LS, and RKZ groups. The four bacterial characteristics (*Enterococcaceae*, *Enterococcus*, *Streptophyta*, and *Viridiplantae*) were richer in the LZ group. *Firmicutes*, *Bacilli, Lactobacillales*, *Lactobacillaceae*, and *Lactobacillus*, were revealed to be five bacterial characteristics significantly richer in the SN group. In the LS group, the bacteria *Clostridia, Gammaproteobacteria, Clostridiales, Clostridiaceae, Enterobacteriaceae, Yersiniaceae*, and *Clostridium* had significantly higher abundance than other groups. A unique flora (21 characteristics) was seen in the RKZ group, including *Negativicutes, Selenomonadales, Selenomonadaceae*, and *Megamonas* (**Figure 8A**, **Figure 9A**). Eight different fungal characteristics were found in the LZ group, including *Leotiomycetes, Agaricomycetes, Eurotiomycetes, Helotiales, Agaricales, Sclerotiniaceae*, *Tausonia*, and *Botrytis*. Fourteen fungal biomarker taxa were found in the SN group, including *Naganishia*, *Mycosphaerella*, *Rhodotorula*, *Alternaria*, and *Sporisorium*. In comparison to other groups, the LS group had considerably higher abundance values for *Sordariomycetes, Saccharomycetes, Hypocreales, Saccharomycetales, Xylariales, Nectriaceae, Debaryomycetaceae, Apiosporaceae, Fusarium, Debaryomyces, Cephalotrichum*, and *Arthrinium*. *Microbotryomycetes, Sporidiobolales, Sporidiobolaceae, Filobasidium*, and *Sporobolomyces* were the fungal biomarkers in the RKZ group (**Figure 7B**, **Figure 9B**).

## Discussion

The IUCN Red List of endangered species classifies the black-necked crane (*G. nigricollis*), one of the 15 crane species found around the world that spends its entire life on the plateau (Liu et al., 2012; Jia et al., 2019), as near endangered. As a flagship species and environmental indicator, crane contributes significantly to preserving the biodiversity of plateau ecosystems (Hou et al., 2021). The primary research areas are population size, distribution, habitat selection, and crane conservation (Kuang et al., 2010; Yang and Zhang, 2014; Li et al., 2022). However, little is understood about the fecal microbiota of cranes. The fecal microbiota plays crucial functions in the host’s health by increasing digestive efficiency, maintaining homeostasis, regulating metabolism, and providing immunological protection (Waite and Taylor, 2015; Gao et al., 2016; Li et al., 2018). Here, the third-generation SMRT sequencing technology was used to analyze and compare the fecal microbial community of cranes raised in four different geographical regions. The goal was to provide a starting point for future research with the aim of disease prevention and species protection.

This study analyzed and compared crane fecal bacterial and fungal communities for the first time. The results showed that there were variations in the fecal microbiota. For example, there was no similar trend between the bacterial and fungal communities, and cranes’ fecal microbial richness and diversity differed significantly. Previous studies have shown that diet is a crucial determinant of the fecal microbial community (Sun et al., 2022). Black-necked cranes in the LS and RKZ were fed potato, barley roots, and *Potentilla anserina L*. in the SN. In contrast, black-necked cranes in the SN mostly foraged *Cyperus rotundus L.*, *Potentilla anserina L.*, and other herbs. Different types of foods impacted how the fecal microbiota colonized (Sun et al., 2022). Meanwhile, some research also showed that long-distance migration primarily affects the bird’s fecal microbiota due to the changes in the habitat environment in the migratory process (Phillips et al., 2018; Wu et al., 2018; San Juan et al., 2020). Other wild animals showed similar results (Barelli et al., 2015; Tzeng et al., 2015; Price et al., 2017). The crane that inhabits the Qinghai–Tibet plateau migrated from several breeding areas to wintering areas. Dietary composition and living environment may have contributed to the changes in fecal microbial composition found in cranes from four different geographical regions.

The wild crane was thought to have a distinct intestinal microbiota because of the varied living environments in the various wintering areas. Only four bacterial and 20 fungal ASVs are shared by four different regions. The results matched PCoA. Distinct crane groups had varying fecal microbial compositions. In addition to food resources, other factors such as altitude, age, sex, and other environmental factors may also contribute to these differences in beta diversity. A few studies found that fecal microbial composition was associated with altitude (Zhang et al., 2016; Li et al., 2018). However, the fecal microbial composition changes in cranes were not correlated with changes in the corresponding altitudes. Due to individual differences, the studies found that the age of the host had a significant impact on fecal microbiota (Bibbo et al., 2016). Meanwhile, sex differences have an impact on intestinal microflora (Capunitan et al., 2020). The fecal microbial communities could be influenced by environmental factors such as climate, fauna, flora, and the host’s age and sex (Song et al., 2020). Therefore, multiple factors contributed to the variations in the fecal microbiota of cranes found in different geographical regions.

Twenty fecal samples were used in our study, identifying 10 bacterial phyla and 9 fungal phyla. The bacterial phyla were primarily made up of Firmicutes and Proteobacteria, and this result was consistent with earlier studies on other birds, including the *bar-headed goose* (Dong et al., 2021), *Whooper Swan* (Wang et al., 2021), *Great Bustard, Common Coot*, and *Common Crane* (Lu et al., 2022). Animals have a large distribution of Firmicutes, essential to hosts in sustaining energy metabolism (Kaoutari et al., 2013) and as a gauge of fecal health (Ley et al., 2006). Crops, vegetation, and animal debris comprised most black-necked crane diets in the overwintering region (Dong et al., 2016). Therefore, the greater relative abundance of Firmicutes may aid in the ability of black-necked cranes to digest and absorb food nutrients and withstand the harsh environment. According to *hooded cranes*, Ascomycota and Basidiomycota comprised the most fungal species (Wu et al., 2022). To break down complex polysaccharides and enhance the ability of the host to absorb nutrients, several Ascomycota members secret large cellulase and hemicellulase (Linton, 2020; Sun et al., 2022). Maintaining fecal ecological balance and function benefits from the abundance of Ascomycota and Basidiomycota (Li et al., 2021). These dominant phyla play vital roles for cranes in maintaining energy metabolism. Regarding genera, the intestinal microbiota of black-necked cranes comprised 15 dominant bacterial genera and 18 dominant fungal genera. A higher percentage of these were *Lactobacillus*, *Megamonas*, and *Enterococcus*, which were widely distributed in other birds and were influential in regulating intestinal health, metabolic capacity, and antimicrobial activity (Gao et al., 2021; Zhao et al., 2022). Remarkably, we also discovered several genera that are opportunistically pathogenic, including *Pseudomonas*, *Pantoe*, *Escherichia*, *Burkholderia*, *Helicobacter*, *Fusarium*, and *Rhodotorula* (Xiang et al., 2019; Wang et al., 2020; Wu et al., 2022). Overall, the data suggested that monitoring the changes in pathogenic species was important for conserving endangered species. We conducted an LEfSe analysis taking into account the different overwintering regions and found a few unique flora at the genus level, such as *Enterococcus*, *Tausonia*, and *Botrytis* in the LZ group; *Lactobacillus*, *Naganishia*, *Mycosphaerella*, *Rhodotorula*, *Alternaria*, and *Sporisorium* in the SN group; *Clostridium, Fusarium, Debaryomyces, Cephalotrichum*, and *Arthrinium* in the LS group; and *Megamonas*, *Fusobacterium*, *Anaerobiospirillum*, *Campylobacter*, *Escherichia*, *Filobasidium*, and *Sporobolomyces* in the RKZ group. Probiotics such as *Enterococcus*, *Megamonas*, *Clostridium*, and *Lactobacillus* have been proven in studies to enhance intestinal microenvironment, metabolic capacity, and antimicrobial activity (Yang et al., 2012; Gao et al., 2021; Zhao et al., 2022). However, *Fusarium* and *Rhodotorula* may be harmful to cranes (Wu et al., 2022). It was shown that intestinal micro-ecology modifies environmental changes (such as diet and altitude) by regulating flora abundance, allowing the host to better adapt to the environment. In this study, 129 bacterial species and 183 fungal species were found, including dominant bacterial species, such as *Campylobacter canadensis*, *Enterococcus faecium*, *Lactobacillus aviaries*, *Fusobacterium mortiferum*, *Escherichia coli*, *Turicibacter* sp. H121, and *Rahnella aquatilis* and dominant fungal species, such as *Mycosphaerella tassiana*, *Naganishia albidosimilis*, and *Naganishia adeliensis*. However, the dominant microbial species per sample was different. Traditional culture methods further explored the function of these species in cranes.

In summary, we first analyzed the intestinal microbial community of cranes using third-generation SMRT sequencing technology. We found the difference in fecal microbiota in cranes in different geographical regions. Factors such as diet, climate, and flora may drive this difference. The community composition of the intestinal microbiota in cranes was accurately analyzed in this study, providing a theoretical basis for identifying and applying functional probiotics, disease prevention, and species protection.

## Data availability statement

The datasets presented in this study can be found in online repositories. The names of the repository/repositories and accession number(s) can be found in the article/**Supplementary Material**.

## Ethics statement

The animal study was approved by The Northeast Forestry University Animal Care Committee reviewed and approved the animal work. The study was conducted in accordance with the local legislation and institutional requirements.

## Author contributions

ZBW designed the conception and design of the research. ZBW, and EHZ collected the samples. ZBW, EHZ, CZ, KR, PS, SM, YT, and JJW performed analysis and interpretation of data and statistical analysis. ZBW and EHZ wrote the first draft of the manuscript. JZM revised the manuscript for important intellectual content. All authors have read and approved the manuscript.
