# Supplementary material for: Comparative analysis of the intestinal microbiota of black−necked cranes (Grus nigricollis) in different wintering areas
Source: Front Cell Infect Microbiol. 2024 Jan 22;13:1302785. doi: 10.3389/fcimb.2023.1302785 (PMC10840423; doi:10.3389/fcimb.2023.1302785)
Supplement: Supplementary file 1 [file DataSheet_1.docx]

# Supplementary Figures


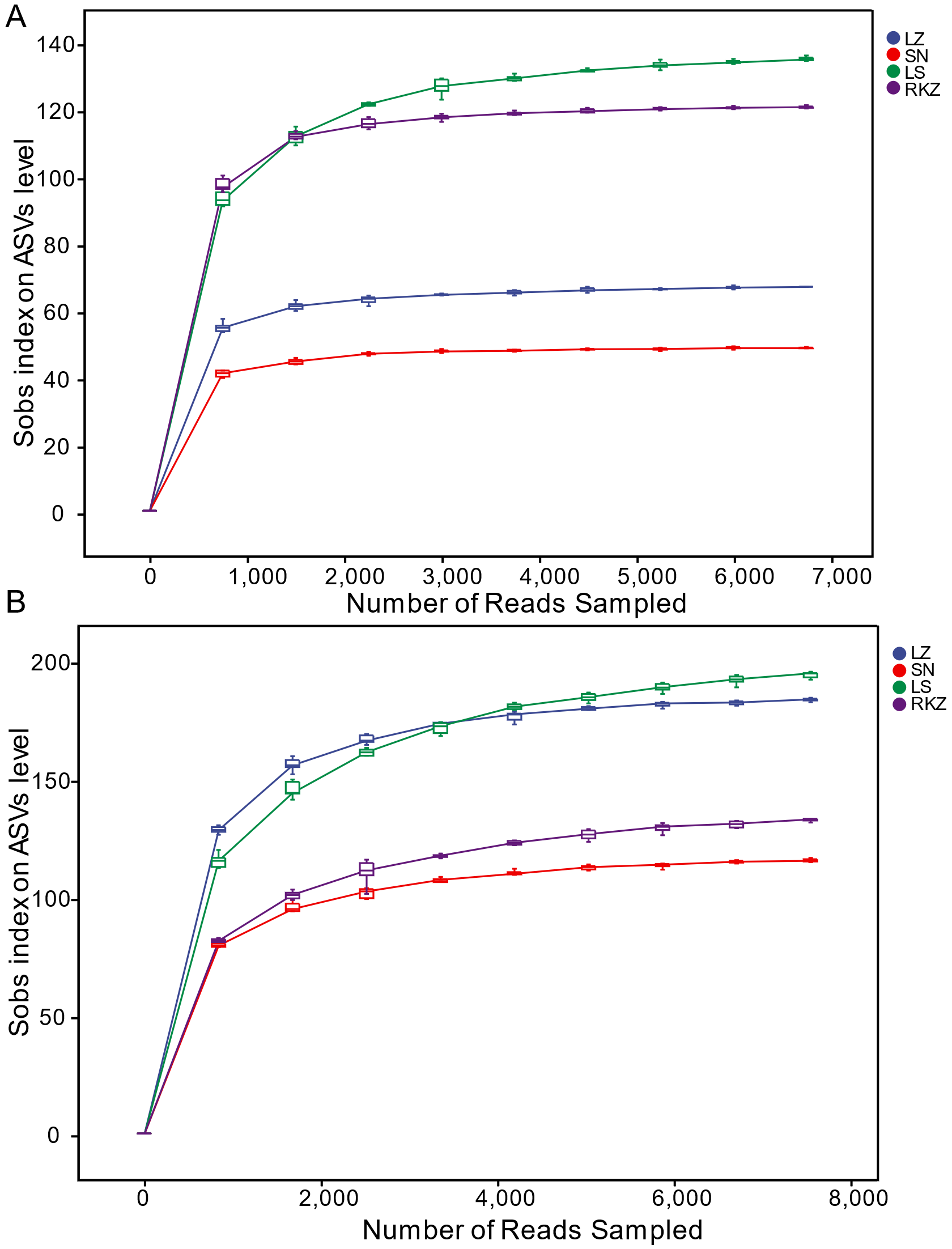


Figure S1. Average rarefaction curve of bacterial (A) and fungal (B) populations of four groups.
